# An enterogenous cyst with atypical pathological findings and chemical meningitis

**DOI:** 10.1186/s40064-016-3677-0

**Published:** 2016-11-21

**Authors:** Lu Wang, Xiaona Chang, Chao Fu, Weidong Yu, Xiaoxuan Fang

**Affiliations:** 1 Department of Neurosurgery, The China-Japan Union Hospital, Jilin University, Changchun, 130033 Jilin People’s Republic of China; 2 The Key Laboratory of Pathobiology, Ministry of Education, Bethune Medical School, Jilin University, Changchun, 130021 Jilin People’s Republic of China

**Keywords:** Enterogenous cyst, Neurenteric cyst, Meningitis, Immunohistochemistry

## Abstract

**Introduction:**

Intracranial enterogenous cysts are rare and mainly occur in the posterior fossa. These cysts are usually extra-axial, midline, anterior to the brainstem, or at the cerebellopontine angle. We report a case of an enterogenous cyst in which diagnosis was difficult because the lesion showed atypical pathologic findings.

**Case presentation:**

A healthy 41-year-old man complained of paroxysmal occipital headaches lasting over a week, with increased severity for 3 days accompanied by slight dizziness and mild nausea. Magnetic resonance imaging showed a cystic lesion between clivus and brainstem. The patient underwent surgery for removal of the lesion via the right-sided far-later approach, and the lesion was resected totally. Although pathologic examinations showed a cyst had a mono-to-multilayered squamous epithelium, which are not accord with typical enterogenous cyst, the diagnosis was finally made based on the presence of basement membrane and immunohistochemical results.

**Discussion and Evaluation:**

To confirm the diagnosis of enterogenous cyst, further pathologic examinations were performed and immunohistochemical characters were summarized. Chemical meningitis, a rare complication of enterogenous cyst, happened in current case. Use a syringe and aspirate the contents before incision might be a procedure to prevent chemical meningitis.

**Conclusions:**

To our knowledge, this is the first report of an enterogenous cyst associated with mono-to-multilayered squamous epithelium. Although during the follow-up time, no recurrence happened, long-term follow-up is needed.

## Background

Enterogenous cysts are rare, benign, congenital lesions, which account for approximately 0.01% of CNS tumors, and commonly locate in the spinal canal, especially at the cervical or upper thoracic level (Gauden et al. 2012; Wang et al. 2011). Intracranial enterogenous cysts are rare and mainly occur in the posterior fossa. These cysts are usually extra-axial, midline, anterior to the brainstem, or at the cerebellopontine angle (Matsumoto et al. 2016). Because the lesion has no characteristic features on radiology, the diagnosis of enterogenous cyst is mainly based on histologic findings (Preece et al. 2006). More rarely the chemical meningitis caused by rupture of a enterogenous cyst in prepontine cistern has been reported. Here, we describe a rare case of an intracranial enterogenous cyst complicated with postoperative chemical meningitis, in which diagnosis was difficult because the lesion showed atypical pathologic results.

## Case presentation

**Fig. 1:**
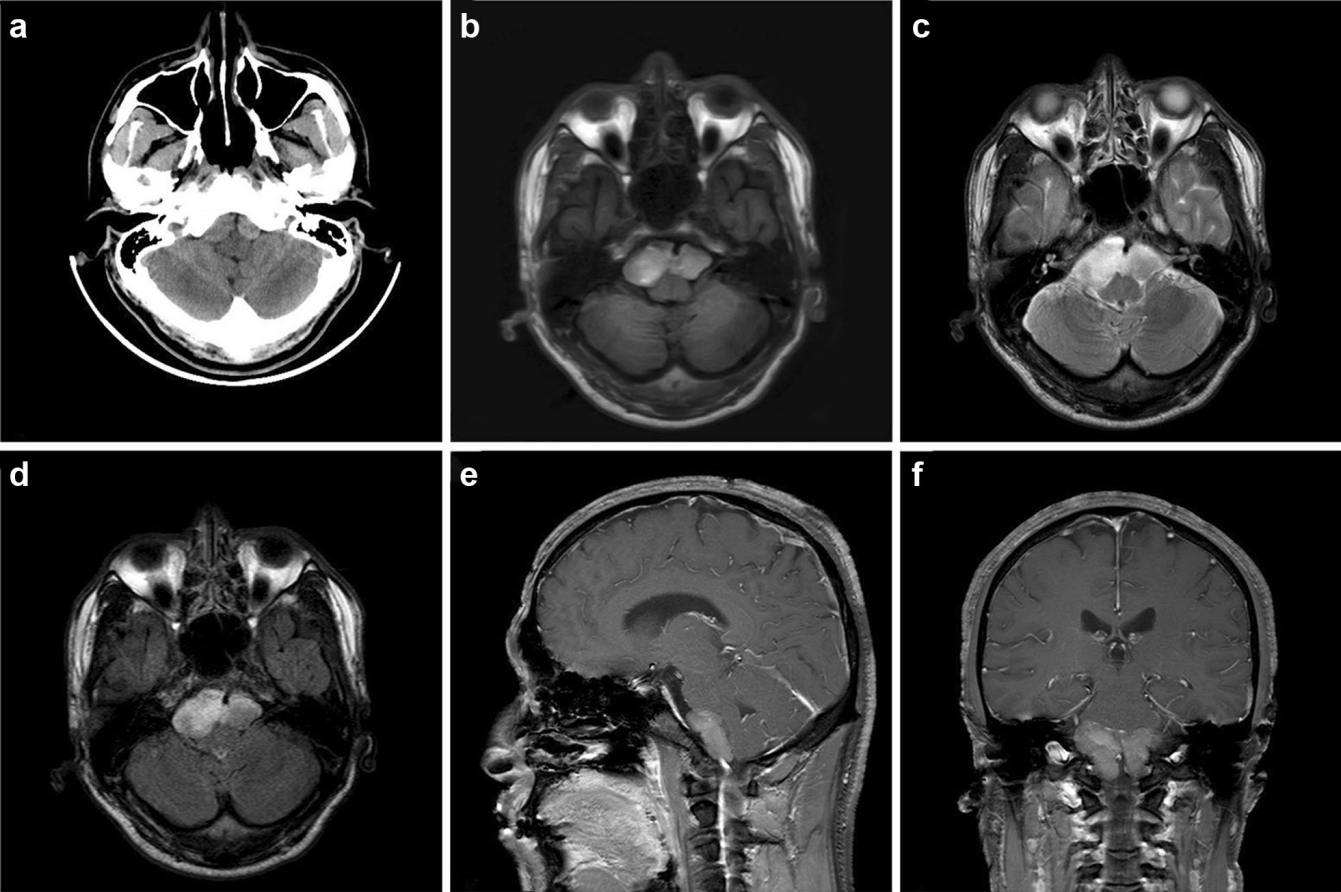
Preoperative computed tomography and magnetic resonance imaging: **a** axial non-contrast computed tomography (CT) scan showing a slight hyperdense lesion lying in the prepontine cistern; **b**, **c** axial T1- and T2-weighted magnetic resonance imaging (MRI) demonstrated a hyperintense mass in the prepontine cistern, the basal artery was enveloped and the brainstem was compressed; **d** axial FLAIR image shows a hyperintense extra-axial cyst compared with CSF; **e**, **f** sagittal and coronal enhanced MRI scans demonstrate a heterogeneously non-enhanced ovoid mass located ventral to pons and medulla

An otherwise healthy 41-year-old man complained of paroxysmal occipital headaches lasting over a week, with increased severity for 3 days accompanied by slight dizziness and mild nausea. These symptoms lasted 10–20 min every time and were exacerbated by postural changes. No coma, seizure, or other signs were reported. Neurological examination showed no evidence of focal deficit. Computer tomography (CT) of brain revealed a hyper-dense lesion in the prepontine cistern (Fig. 1a), and magnetic resonance imaging (MRI) showed an oval cystic lesion located between clivus and brainstem, measuring 4.6 cm × 1.6 cm. The cyst contents were inhomogeneous hyper-intense on T1-weighted and T2-weighted images (Fig. 1b, c). The fluid-attenuated inversion recovery (FLAIR) sequences showed cyst hyperintensity to CSF (Fig. 1d). Gadolinium administration caused no enhancement of the cyst wall and contents (Fig. 1e, f). The patient underwent surgery for removal of the lesion via the right-sided far-later approach. The wall of the lesion was semi-transparent, white-grayish and fragile. Disruption of the cyst wall released gelatinous and yellowish cystic contents slowly. After aspiration of the contents, the lesion was dissected away from the surrounding neurovascular structures and removed totally. The postoperative MRI revealed that the lesion disappeared completely. Postoperatively, the patient manifested fever, headache, nausea, and positive signs of meningeal irritation. Cerebrospinal fluid (CSF) specimens were collected by lumbar punctures and laboratory results showed polymorphonuclear pleocytosis, slightly higher protein and negative culture results. Repeated lumber punctures were performed and a continuous lumbar subarachnoid drainage was retained for 3 days. The leukocyte count in the CSF decreased gradually, accompanied with a gradual normalization of body temperature. Seven days after operation, the symptoms caused by meningeal irritation were relieved, and the patient was discharged uneventfully. The patient remains asymptomatic 6 months after the operation and the follow-up MRI demonstrated no recurrence of the cyst.

## Discussion

Enterogenous cysts have been defined as benign cystic lesions that are lined by mucin-secreting epithelium similar to those found in the gastrointestinal tract. Wilkins et al. proposed a classification system for intraspinal enterogenous cysts (Types A–C) based on histological features (Wilkins and Odom 1976):

Type A cysts have an epithelial lining composed of pseudostratified cuboidal or columnar epithelial cells mimicking the respiratory or gastrointestinal epithelium.

Type B cysts in addition to the above structure, may be arranged in complex invaginations and have associated glands producing mucinous or serous fluid. These cysts may be composed of a range of associated tissue including smooth muscle, striated muscle, fat, cartilage, bone, elastic fibers, lymphoid tissue, nerve fibers, ganglion cells, or Vater Pacini corpuscles.

Type C cysts, in addition to the findings in Type B, may be associated with glial elements such as ependymal cells of the wall.

**Fig. 2:**
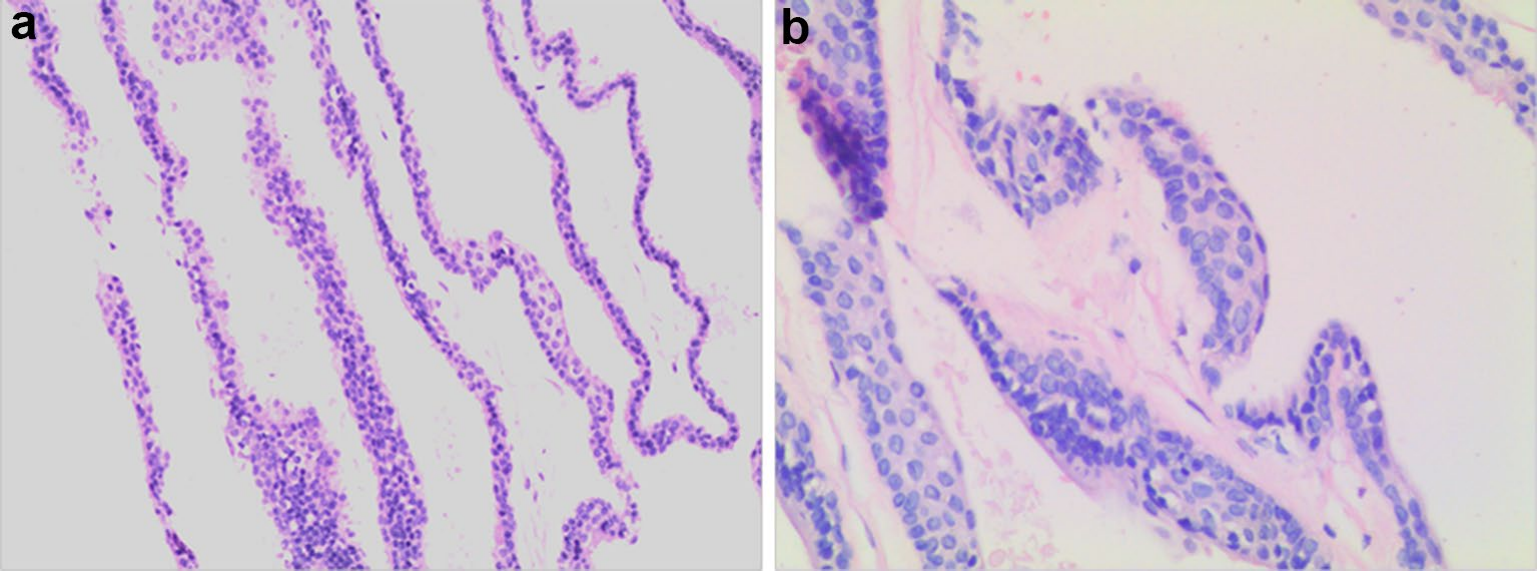
Photomicrographs of surgical specimens stained with hematoxylin and eosin. The cyst wall is lined with mono-to-multilayered flattened, non-ciliated epithelial cells supported by glial tissue. **a** Original magnification 100, **b** original magnification 200

**Fig. 3:**
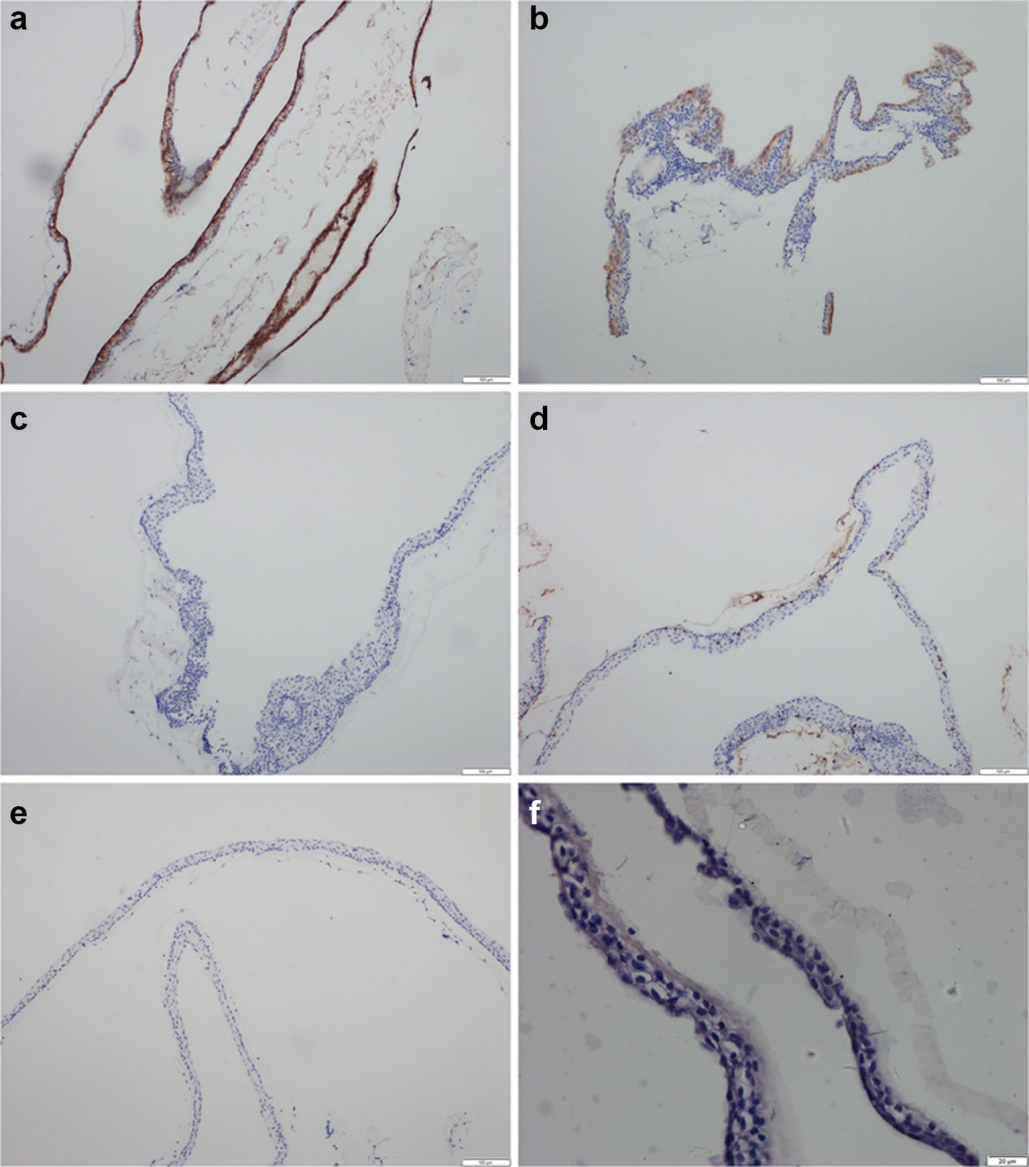
Immunohistochemistry of the cyst. **a** Epithelial membrane antigen immunostaining showed a positive reaction in the epithelium (original magnification 400); **b** carcinoembryonic antigen stained positive in the epithelium (original magnification 400); **c** neuron-specific enolase stained negative in the epithelium (original magnification 400); **d** vimentin immunostaining was negative in the epithelium (original magnification 400); **e** immunostaining for glial fibrillary acidic protein expression was negative in the epithelium (original magnification 400); **f** periodic acid–Schiff staining shows a positive reaction in the basement membrane (original magnification 400)

However, there are no classification exists for intracranial enterogenous cysts, mainly because of their rarity. In current case, we were unable to identify the correct histological type. The atypical pathologic results were major challenge in arriving at a definitive diagnosis. In our case, routine hematoxylin and eosin staining showed that the cyst wall was lined with mono-to-multilayered, non-ciliated, squamous epithelium instead of single or pseudostratified layer of ciliated or non-ciliated, columnar or cuboidal epithelium (Fig. 2a, b). To confirm the diagnosis of enterogenous cyst, further examinations were performed. The epithelium tested positive for epithelial membrane antigen (Fig. 3a) and carcinoembryonic antigen (Fig. 3b). Further, it was immunonegative for neuron-specific enolase (Fig. 3c), vimentin (Fig. 3d) and glial fibrillary acidic protein (Fig. 3e). With periodic acid–Schiff staining, a positive reaction was found in the basement membrane (Fig. 3f). Immunohistochemical features of intracranial cystic lesions are summarized in Table 1. Colloid and enterogenous cysts share similar immunohistochemical manifestation. While intracranial enterogenous cysts are mostly located near the midline in the posterior fossa, colloid cysts tend to occur within the third ventricle (Gauden et al. 2012; Preece et al. 2006). Based on the results of immunocytochemistry and cyst location, we arrived at a diagnosis of enterogenous cyst of prepontine cistern. Although squamous metaplasia (Preece et al. 2006), and cerebellar enterogenous cyst lined with a single layer of squamous epithelial cell have been reported (Matsumoto et al. 2016), to the best of our knowledge this is the first case presenting with an enterogenous cyst lined with mono-to-multilayered squamous epithelium.

**Table 1:** Immunohistochemical findings for intracranial cystic lesion

Lesion type	EMA	CEA	Vimentin	GFAP	NSE
Enterogenous cyst	+	+	–	–	–
Arachnoid cyst	+	–	+	–	+
Ependymal cyst	+	–	–	+	+
Choroidplexus cyst	–	–	+	–	+
Colloid cyst	+	+	–	–	–

Base on lumbar CSF examinations, infection was excluded and chemical meningitis was diagnosed, which was caused by cyst leakage intraoperatively (Gauden et al. 2012; Wang et al. 2011). Elevated CSF protein levels supported this hypothesis. Postoperative chemical meningitis has been rarely reported for intracranial enterogenous cysts (Wang et al. 2011). To prevent postoperative chemical meningitis, several authors recommend puncture of the cysts using a syringe and aspiration of the contents before incision (Wang et al. 2011). Although complete surgical excision improved the prognosis, based on previous studies suggesting recurrence between 4 months and 14 years (Preece et al. 2006), this case warranted long-term clinical and radiological follow-up.

## Conclusions

To our best knowledge, we present the first case of intracranial enterogenous cyst associated with mono-to-multilayered squamous epithelium. Because of the lack of radiographic features and complicated appearance in light microscopy, histopathological examinations play an important role in diagnosis. In our case, during 6-month follow-up, the patient remained neurologically intact without radiographical evidence of cyst recurrence. Nevertheless, long-term follow-up is advised to detect the recurrence as early as possible.

## Abbreviations

CT: computer tomography
MRI: magnetic resonance imaging
FLAIR: the fluid-attenuated inversion recovery
CSF: cerebrospinal fluid
